# Effect of very preterm delivery on outcomes following in utero spina bifida repair

**DOI:** 10.1002/pmf2.70027

**Published:** 2025-04-30

**Authors:** Michael V. Zaretsky, Jagruti Anadkat, Kelly A. Bennett, Yair J. Blumenfeld, Chris Buchanan, Caitlin Clifford, Timothy Crombleholme, Samer Elbabaa, Stephen P. Emery, William H. Goodnight, Hanmin Lee, Joseph B. Lillegard, Foong‐Yen Lim, Francois I. Luks, Virginia Lijewski, Jena L. Miller, Ueli Moehrlen, Julie Moldenhauer, Anita J. Moon‐Grady, Mauro Schenone, Daniel W. Skupski, Aimen F. Shaaban, Kuojen Tsao, Tim Van Mieghem, Amy J. Wagner

**Affiliations:** ^1^ Colorado Fetal Care Center University of Colorado School of Medicine Aurora Colorado USA; ^2^ The Fetal Care Center Washington University School of Medicine St. Louis Missouri USA; ^3^ The Fetal Center at Venderbilt Vanderbilt University Nashville Tennessee USA; ^4^ Lucile Packard Children's Hospital Stanford University School of Medicine Palo Alto California USA; ^5^ St. Louis Fetal Care Institute Saint Louis University School of Medicine St. Louis Missouri USA; ^6^ Fetal Diagnosis and Treatment Center University of Michigan Ann Arbor Michigan USA; ^7^ Connecticut Children's Fetal Care Center University of Connecticut School of Medicine Hartford Connecticut USA; ^8^ Orlando Health Center for Fetal Care Orlando Health Arnold Palmer Hospital for Children Orlando Florida USA; ^9^ Magee‐Womens Hospital of the University of Pittsburgh Medical Center University of Pittsburgh Pittsburgh Pennsylvania USA; ^10^ UNC Collaborative for Maternal and Infant Health University of North Carolina at Chapel Hill Chapel Hill North Carolina USA; ^11^ UCSF BCH Fetal Treatment Center University of California San Francisco San Francisco California USA; ^12^ Midwest Fetal Care Center Children's Minnesota Minneapolis Minnesota USA; ^13^ Cincinnati Children's Fetal Care Center Cincinnati Children's Hospital Cincinnati Ohio USA; ^14^ Fetal Treatment Program of New England Hasbro's Children's Hospital Providence Rhode Island USA; ^15^ Johns Hopkins Center for Fetal Therapy Johns Hopkins University School of Medicine Baltimore Maryland USA; ^16^ Children's Hospital Zurich Zurich Switzerland; ^17^ Richard D. Wood Jr. Center for Fetal Diagnosis and Treatment Children's Hospital of Philadelphia Philadelphia Pennsylvania USA; ^18^ UCSF BSH Fetal Treatment Center University of California San Francisco San Francisco California USA; ^19^ Mayo Clinic Children's Center Mayo Clinic Rochester Minnesota USA; ^20^ Fetal Care Center Weill‐Cornell University New York New York USA; ^21^ The Chicago Institute for Fetal Health Ann & Robert H. Lurie Children's Hospital of Chicago Chicago Illinois USA; ^22^ UT Health Houston Fetal Center University of Texas Health Science Center Houston Texas USA; ^23^ Ontario Fetal Centre Fetal Medicine Unit, Mount Sinai Hospital and University of Toronto Toronto Ontario Canada; ^24^ Fetal Concerns Center Children's Hospital of Wisconsin Milwaukee Wisconsin USA

**Keywords:** fetal surgery, neonatal outcomes, preterm birth, spina bifida, ventriculoperitoneal shunt

## Abstract

**Objective:**

In utero fetal spina bifida repair decreases the risk for cerebrospinal fluid (CSF) diversion including ventriculoperitoneal (VP) shunt placement, reverses hindbrain herniation, and improves motor function with a higher likelihood of independent ambulation. Unfortunately, in utero surgery can be complicated by preterm delivery. The goal of this study was to compare pregnancy and neonatal outcomes between individuals who delivered very preterm, and those who delivered later in gestation following in utero spina bifida repair.

**Methods:**

Data from the North American Fetal Therapy Network Fetal Myelomeningocele Consortium Registry collected from 2011 to 2024 were analyzed. Three cohorts of patients were categorized by gestational age at delivery and compared: <30 weeks, 30–34 weeks, and >34 weeks. Comparisons between groups for maternal and delivery outcomes were completed for the entire cohort and then analyzed for those with available CSF diversion data up to 1 year of life. Chi‐square or Fisher's exact tests were used to compare categorical variables, and Kruskal–Wallis tests were used to determine if there are significant differences between the distributions of the three groups. A *p*‐value <0.05 was considered significant.

**Results:**

A total of 1213 patients were analyzed (<30 weeks, *n* = 111 [9.2%]; 30–34 weeks, *n* = 323 [26.6%]; >34 weeks, *n* = 779 [64.2%]). Overall fetal and neonatal mortality was 2.2%, 85% of which occurred in the <30‐week cohort, where perinatal mortality was 21%. Latency from fetal surgery to delivery averaged 2.1 weeks for the <30‐week group. Rates of abruption, chorioamnionitis, and spontaneous labor were higher in the <30‐week group compared to later gestational age cohorts. Neonatal morbidity was also significantly higher in the <30‐week cohort for sepsis, apnea, respiratory distress, patent ductus arteriosus, intraventricular hemorrhage, and periventricular leukomalacia in the <30‐week cohort. CSF diversion data were available for 677 neonates (<30 weeks, *n* = 53; 30–34 weeks, *n* = 186; >34 weeks, *n* = 438). There was no significant difference in VP shunt placement between the different gestational age groups.

**Conclusion:**

Very preterm birth following in utero spina bifida repair results in significant perinatal mortality and neonatal morbidity. Delivery prior to 30 weeks of gestation occurs in 9% of patients after in utero spina bifida repair, resulting in a 21% perinatal mortality rate. However, even among those who delivered very preterm, a reduction in VP shunt rates, a benefit of in utero repair, appears to be preserved. Further data are warranted to assess the impact of prematurity following in utero surgery on ambulation and developmental outcomes.

## 1 INTRODUCTION

In utero myelomeningocele repair decreases the risk for cerebrospinal fluid (CSF) diversion including ventriculoperitoneal (VP) shunt placement, reverses hindbrain herniation, and improves motor function with a higher likelihood of independent ambulation for those born with spina bifida [1, 2]. Because of these proven benefits, both in utero surgical repair and neonatal repair options are offered as standard of care for prenatally diagnosed spina bifida. Pregnant patients must weigh the benefits of in utero repair against the risks of potentially significant maternal and fetal complications resulting from fetal surgery [3, 4].

Delivering prior to 30 weeks of gestation is a major complication that occurs in approximately 13% of cases [1]. The risk of fetal and neonatal mortality from prematurity induced by prenatal surgery is balanced against a similar rate of mortality from complications from spina bifida following standard neonatal repair [5]. Premature birth and its accompanying morbidity and mortality accounts for a significant component of presurgical counseling for those considering prenatal intervention. Outcome data for those who delivered very preterm following in utero spina bifida repair have been limited to a small number of patients in existing studies, and the paucity of information regarding the maternal and neonatal outcomes makes counseling families challenging.

The objective of this study was to compare pregnancy and neonatal outcomes including CSF diversion rates between those who delivered very preterm following in utero surgery and those who delivered at later gestational ages (GAs) to inform prenatal counseling and provide critical data for prospective surgical candidates considering in utero versus neonatal repair options.

## 2 MATERIALS AND METHODS

The North American Fetal Therapy Network (NAFTNet) is a voluntary association of 54 medical centers in North America and Europe that performs advanced in utero fetal therapeutic procedures. The NAFTNet Fetal Myelomeningocele (fMMC) Consortium registry has accrued significant data on both maternal and neonatal outcomes since its inception in 2012, including a growing number of cases that delivered prior to 30 weeks [6, 7]. The fMMC Consortium registry, supported by NAFTNet, is a prospective observational registry. This registry aims to track maternal and neonatal outcomes after maternal–fetal surgery for fMMC done after the completion of the Management of Myelomeningocele Study (MOMS) trial. Institutions performing fMMC surgery were invited to participate, with each site responsible for enrolling eligible patients and entering and ensuring data accuracy. The coordinating center obtained institutional review board (IRB) approval, with individual sites securing their own IRB approval to collect data prospectively from consenting patients.

Retrospective data were gathered from maternal and neonatal electronic health records (EHRs), in compliance with Health Insurance Portability and Accountability Act regulations [8]. Study data are collected and managed using the Research Electronic Data Capture (REDCap) electronic data capture tool. REDCap is a secure, web‐based application designed to support data capture for research studies, providing an intuitive interface for validated data entry, audit trails for tracking data manipulation and export procedures, automated export procedures for seamless data downloads to common statistical packages, and procedures for importing data from external sources [9]. De‐identified data from the registry were provided to the investigator for specific study questions. The investigators were blinded to individual clinical site and all patient‐identifying information. Center‐specific data for surgical technique, including open versus fetoscopic repair, experience, and outcomes, are not available. The preponderance of surgery was performed through open fetal repair with a low percentage of cases performed through a fetoscopic approach. The data used for this analysis include patients who began evaluation for fMMC on or after December 15, 2010.

Three cohorts of patients were categorized by GA of delivery and compared: <30, 30–34, and >34 weeks. In this study, we defined very preterm birth as delivery occurring at <30 weeks gestation. While the World Health Organization (WHO) defines very preterm birth as birth between 28 and <32 weeks, we adopted the threshold of <30 weeks to ensure consistency with definitions utilized in prior reports. In the MOMS trial, post‐MOMS experience as well as subsequent studies comparing prenatal repair techniques, these three cohorts were utilized for comparison. To ensure adequate numbers were available for analysis, the <30‐week cohort was not divided further into a <28‐week cohort, although total deliveries occurring <28 weeks are provided. Comparisons between groups for maternal and delivery outcomes were completed for the entire cohort and then analyzed for those with available CSF diversion data up to 1 year of life. The registry currently does not contain significant data regarding ambulation or neurodevelopmental outcomes in the very preterm birth cohort. Typical morbidities of prematurity, including respiratory distress syndrome, apnea, patent ductus arteriosus, sepsis, necrotizing enterocolitis, intraventricular hemorrhage, and periventricular leukomalacia, were assessed. Perinatal mortality included stillbirth and neonatal mortality to day of life 28 and was further defined by timing of demise.

Chi‐square or Fisher's exact tests were used to compare categorical variables, and Kruskal–Wallis tests was used to compare two or more independent groups to determine if there are statistically significant differences between their medians or distributions with *p*‐values <0.05 considered significant. The analysis for this paper was generated using SAS software. Copyright 2024 SAS Institute Inc. SAS and all other SAS Institute Inc. product or service names are registered trademarks or trademarks of SAS Institute Inc.

## 3 RESULTS

A total of 1213 patients were available for analysis in the registry (<30 weeks, *n* = 111 [9.2%]; 30–34 weeks, *n* = 323 [26.6%]; >34 weeks, *n* = 779 [64.2%]). Of the 111 patients delivering prior to 30 weeks, 70 (63%) of infants were born at <28 weeks. A total of 1084 (89%) patients had an open fetal repair, and 72 (6%) patients had a fetoscopic repair documented. The median GA at delivery was higher for patients who received an open procedure compared to patients who received a fetoscopic procedure (35.4 vs. 33.7 weeks, *p* < 0.01), and infants born at <30 weeks were more likely to be born to patients who received a fetoscopic repair (11.8%) compared to infants born between 30 and 34 weeks (8.9%) or >34 weeks (4.2%) (*p* < 0.01). CSF diversion data, up to 1 year of life, were available for 677 patients (<30 weeks, *n* = 53 [7.8%]; 30–34 weeks, *n* = 186 [27.5%]; >34 weeks, *n* = 438 [64.7%]). Only 17 of the 72 patients who underwent fetoscopic repair have CSF diversion data available in the registry with only two within the <30‐week cohort. Baseline characteristics were similar between GA cohorts (Tables 1 and 2), except for median body mass index (BMI) for those with available CSF diversion data (*p* = 0.03). In the <30‐week cohort, the median latency from surgery to delivery was 2.1 weeks with a median GA of delivery at 27.1 weeks (Table 3). Overall perinatal mortality was 2.2%, with 85% occurring within the <30‐week cohort, in which there was a 21% perinatal mortality rate. The median GA at delivery in the <30‐week cohort resulting in death was 25.8 weeks. Stillbirth occurred in 10.1% and neonatal death occurred in 11.7%. Maternal complications were significantly higher for those in the <30‐week cohort, with higher rates of placental abruption, chorioamnionitis, and need for blood transfusion compared to later GA cohorts. Chorioamniotic membrane separation, oligohydramnios and spontaneous membrane rupture developed at higher rates in the 30–34‐week cohort. In the later GA cohorts, there was also a higher rate of a thin hysterotomy scar at time of delivery. Neonatal morbidity was also significantly higher for sepsis, apnea, respiratory distress, patent ductus arteriosus, intraventricular hemorrhage, and periventricular leukomalacia in the <30‐week cohort (Tables 3 and 4). The median neonatal intensive care length of stay was 77 days (42–94 days) in the <30‐week group compared to 32 days (20.5–48.5 days) for the 30–34‐week group and 10 days (6–15 days) for the >34‐week group.

**TABLE 1:** Baseline Characteristics for the entire cohort.

Baseline characteristics entire cohort	Overall, *N* = 1213	Gestational age at delivery <30 weeks (*n* = 111)	Gestational age at delivery 30–34 weeks (*n* = 323)	Gestational age at delivery >34 weeks (*n* = 779)	
Characteristic	*n* (%)	*n* (%)	*n* (%)	*n* (%)	*p*‐value
Fetal sex					0.16
Male	582 (49.2)	57 (51.8)	141 (44.6)	384 (50.7)	
Female	602 (50.8)	53 (48.2)	175 (55.4)	374 (49.3)	
Race					0.12
White	956 (84.8)	85 (83.3)	249 (85.6)	622 (84.6)	
Black	39 (3.5)	2 (2.0)	5 (1.7)	32 (4.4)	
Hispanic	101 (9.0)	13 (12.8)	26 (8.9)	62 (8.4)	
Asian	10 (0.9)	1 (1.0)	1 (0.3)	8 (1.1)	
Other	22 (2.0)	1 (1.0)	10 (3.4)	11 (1.5)	
Marital status					0.22
Single	126 (10.6)	14 (12.8)	2 (13.4)	70 (9.2)	
Married	917 (77.4)	83 (76.2)	240 (76.4)	594 (78.0)	
Single living with partner	142 (12.0)	12 (11.0)	32 (10.2)	98 (12.9)	
Age at estimated due date (median IQR)	30 (26‐34)	29 (26‐33)	30 (26‐33)	30 (27‐34)	0.16
Body mass index (median IQR)	27.1 (23.8‐31.5)	26.2 (23.3‐30.6)	27.9 (24.2‐32.0)	27.1 (23.7‐31.2)	0.11
Nulliparous	465 (38.4)	40 (36.0)	129 (40.0)	296 (38.1)	0.73
Current Smoker	61 (5.1)	11 (10.2)	18 (5.7)	32 (4.2)	
Prior uterine surgery	189 (15.8)	14 (13.1)	51 (16.0)	124 (16.1)	
Anterior placenta	606 (50.0)	57 (51.4)	157 (48.6)	392 (50.3)	0.83
Lesion level on ultrasonography					0.84
Thoracic	36 (3.0)	2 (1.9)	8 (2.5)	26 (3.4)	
L1–L2	241 (20.0)	22 (20.4)	68 (21.2)	151 (19.4)	
L3–L4	676 (56.1)	57 (52.9)	177 (55.1)	442 (56.9)	
L5–S1	249 (20.7)	27 (25.0)	66 (20.6)	156 (20.1)	
Club foot	115 (9.5)	8 (7.2)	30 (9.3)	77 (10.0)	0.66

Abbreviation: IQR, interquartile range.

**TABLE 2:** Baseline characteristics for available cerebrospinal fluid (CSF) diversion data.

Baseline characteristics for available CSF diversion data	Overall, *N* = 677	Gestational age at delivery <30 weeks (*n* = 53)	Gestational age at delivery 30–34 weeks (*n* = 186)	Gestational age at delivery >34 weeks (*n* = 438)	
	*n* (%)	*n* (%)	*n* (%)	*n* (%)	*p*‐value
**Maternal characteristics**					
Fetal sex					0.18
Male	333 (49.6)	27 (50.9)	81 (43.8)	225 (51.8)	
Female	339 (50.4)	26 (49.1)	104 (56.2)	209 (48.2)	
Race					0.71
White	548 (84.4)	44 (84.6)	149 (87.1)	355 (83.3)	
Black	23 (3.5)	1 (1.9)	3 (1.8)	19 (4.5)	
Hispanic	61 (9.4)	5 (9.6)	14 (8.2)	42 (9.9)	
Asian	7 (1.1)	1 (1.9)	1 (0.6)	5 (1.2)	
Other	10 (1.5)	1 (1.9)	4 (2.3)	5 (1.2)	
Marital status					0.36
Single	66 (10.0)	5 (9.6)	23 (12.7)	38 (8.9)	
Married	532 (80.5)	39 (75.0)	143 (79.0)	350 (81.8)	
Single living with partner	63 (9.4)	8 (15.4)	15 (8.3)	40 (9.4)	
Age at estimated due date (median, IQR)	29 (26‐33)	28 (26‐32)	29 (26‐33)	30 (26‐33)	0.15
Body mass index (median, IQR)	27 (24‐31)	26 (24‐30)	28 (25‐32)	27 (24‐31)	0.03
Nulliparous	256 (37.9)	23 (43.4)	73 (39.3)	160 (36.6)	0.57
Current smoker	32 (4.8)	3 (5.7)	9 (4.8)	20 (4.6)	0.94
Prior uterine surgery	101 (15.1)	7 (13.5)	26 (14.2)	68 (15.6)	0.85
Anterior placenta	329 (48.6)	31 (58.5)	95 (51.1)	203 (46.4)	0.18
Lesion level on ultrasonography					0.87
Thoracic	20 (3.0)	2 (3.9)	4 (2.2)	14 (3.2)	
L1–L2	147 (21.8)	14 (26.9)	44 (23.7)	89 (20.4)	
L3–L4	392 (58.1)	28 (53.9)	105 (56.4)	259 (59.3)	
L5–S1	116 (17.2)	8 (15.4)	33 (17.7)	75 (17.2)	
Club foot	110 (16.3)	8 (15.1)	29 (15.6)	73 (16.7)	0.92

Abbreviation: IQR, interquartile range.

**TABLE 3:** Maternal/pregnancy and neonatal outcomes for the entire cohort.

Maternal and neonatal outcomes entire cohort	Overall, *N* = 1213	Gestational age at delivery <30 weeks (*n* = 111)	Gestational age at delivery 30–34 weeks (*n* = 323)	Gestational age at delivery >34 weeks (*n* = 779)	
	*n* (%)	*n* (%)	*n* (%)	*n* (%)	*p*‐value
**Maternal/pregnancy outcomes**					
Chorioamniotic membrane separation	173 (14.3)	17 (15.3)	77 (23.8)	79 (10.1)	<0.01
Pulmonary edema	18 (1.5)	0 (0.0)	4 (1.2)	14 (1.8)	0.31
Oligohydramnios	200 (16.5)	12 (10.8)	77 (23.8)	111 (14.3)	<0.01
Placental abruption	54 (4.5)	13 (11.7)	27 (8.4)	14 (1.8)	<0.01
Gestational diabetes	86 (7.1)	5 (4.5)	24 (7.4)	57 (7.3)	0.54
Chorioamnionitis	65 (5.4)	25 (22.5)	27 (8.4)	13 (1.7)	<0.01
Preeclampsia or gestational hypertension	356 (29.4)	32 (28.8)	93 (28.8)	231 (29.7)	0.95
Spontaneous membrane rupture	382 (31.5)	52 (46.9)	190 (58.8)	140 (18.0)	<0.01
Spontaneous labor	283 (23.3)	50 (45.1)	114 (35.3)	119 (15.3)	<0.01
Blood transfusion at delivery	13 (1.1)	5 (4.5)	5 (1.6)	3 (0.4)	<0.01
Status of hysterotomy at delivery					<0.01
Intact	731 (69.7)	81 (90.0)	204 (75.8)	446 (64.6)	<0.01
Thin	257 (24.5)	6 (6.7)	54 (20.1)	197 (28.6)	<0.01
Focal or area of dehiscence	58 (5.5)	3 (3.3)	11 (4.1)	44 (6.4)	0.25
Complete dehiscence	3 (0.3)	0 (0.0)	0 (0.0)	3 (0.4)	0.46
Stillbirth or neonatal death to day of life 28	27 (2.2)	23 (20.7)	3 (0.9)	1 (0.1)	<0.01
Neonatal death	15 (1.2)	13 (11.7)	1 (0.3)	1 (0.1)	<0.01
Birth					<0.01
Liveborn	1182 (98.8)	98 (89.9)	317 (99.1)	767 (99.9)	
Stillborn	15 (1.3)	11 (10.1)	3 (0.9)	1 (0.1)	
Gestational age at surgery (median, IQR)	24.7 (24.0–25.3)	24.9 (24.0–25.4)	24.6 (23.9–25.5)	24.7 (24.0–25.3)	0.12
Gestational age at delivery (median, IQR)	35.3 (33.3–36.9)	27.1 (25.9–28.9)	32.9 (32.6–33.7)	36.2 (35.4–37.0)	<0.01
Gestational age at death (median, IQR)	25.9 (24.7–28.7)	25.8 (24.6–26.3)	31.7 (30.1–33.9)	37.4 (37.4–37.4)	<0.01
Latency (GA at delivery—GA at surgery)	10.7 (8.4–12.0)	2.1 (0.9–4.2)	8.1 (7.0–9.3)	11.7 (10.7–12.4)	<0.01
**Neonatal outcomes**					
Birthweight (g, median, IQR)	2500 (2020–2870)	980 (800–1200)	2003 (1700–2220)	2750 (2490–2990)	<0.01
Dehiscence at lesion repair site					0.45
Intact	966 (93.4)	78 (93.9)	243 (90.7)	645 (92.8)	
Dehiscence	80 (7.7)	5 (6.0)	25 (9.3)	50 (7.2)	
Apnea	334 (27.5)	72 (64.9)	148 (45.8)	114 (14.6)	<0.01
Respiratory distress syndrome	252 (20.8)	63 (56.8)	104 (32.2)	85 (10.9)	<0.01
Pneumothorax	28 (2.3)	4 (3.6)	12 (4.0)	11 (1.4)	0.02
Patient ductus arteriosus	52 (4.3)	32 (28.8)	13 (4.0)	7 (1.0)	<0.01
Sepsis	43 (3.5)	18 (16.2)	12 (3.7)	13 (1.7)	<0.01
Necrotizing enterocolitis	6 (0.5)	1 (1.0)	3 (1.0)	2 (0.3)	0.29
Intraventricular hemorrhage	21 (1.7)	12 (10.8)	3 (0.9)	6 (0.8)	<0.01
Periventricular leukomalacia	17 (1.4)	4 (3.6)	9 (2.8)	4 (0.5)	<0.01
Foot deformity	306 (25.2)	24 (21.6)	84 (26.0)	198 (25.4)	0.64

Abbreviation: GA, gestational age; IQR, interquartile range.

**TABLE 4:** Outcomes for those with cerebrospinal fluid (CSF) diversion data.

Maternal and neonatal outcomes for available CSF diversion data	Overall, *N* = 677	Gestational age at delivery <30 weeks (*n* = 53)	Gestational age at delivery 30–34 weeks (*n* = 186)	Gestational age at delivery >34 weeks (*n* = 438)	
	*n* (%)	*n* (%)	*n* (%)	*n* (%)	*p*‐value
**Maternal/pregnancy outcomes**					
Chorioamniotic membrane separation	99 (14.6)	9 (17.0)	47 (25.3)	43 (9.8)	<0.01
Pulmonary edema	8 (1.2)	0 (0.0)	2 (1.1)	6 (1.4)	0.68
Oligohydramnios	103 (15.2)	10 (18.9)	44 (23.7)	49 (11.2)	<0.01
Placental abruption	30 (4.4)	8 (15.1)	13 (7.0)	9 (2.1)	<0.01
Gestational diabetes	32 (4.7)	3 (5.7)	7 (3.8)	22 (5.0)	0.75
Chorioamnionitis	25 (3.7)	11 (20.8)	10 (5.4)	4 (0.9)	<0.01
Preeclampsia or gestational hypertension	262 (38.7)	20 (37.7)	70 (37.6)	172 (39.3)	0.92
Spontaneous membrane rupture	217 (32.1)	30 (56.6)	111 (59.7)	76 (17.4)	<0.01
Spontaneous labor	186 (27.5)	30 (56.6)	75 (40.3)	81 (18.5)	<0.01
Blood transfusion at delivery	10 (1.5)	4 (7.6)	4 (2.2)	2 (0.5)	<0.01
Status of hysterotomy at delivery					0.25
Intact	471 (74.3)	41 (89.1)	128 (75.7)	302 (72.1)	
Thin	128 (20.2)	3 (6.5)	34 (20.1)	91 (21.7)	
Focal or area of dehiscence	34 (5.4)	2 (4.4)	7 (4.1)	25 (6.0)	
Complete dehiscence	1 (0.2)	0 (0.0)	0 (0.0)	1 (0.2)	
Stillbirth or neonatal death to day of life 28	0 (0.0)	0 (0.0)	0 (0.0)	0 (0.0)	
Neonatal death	1 (0.2)	0 (0.0)	0 (0.0)	1 (0.2)	0.76
Gestational age at delivery (median, IQR)	35.3 (33.4)	27.4 (26.6–29.1)	32.9 (31.6–33.7)	36.6 (35.4–37.0)	<0.01
Latency (GA at delivery—GA at surgery) (median, IQR)	11.0 (8.9–12.1)	3.3 (1.4–4.6)	8.4 (7.2–9.6)	11.9 (11.1–12.7)	<0.01
**Neonatal/infant outcomes**					
Shunt placement	310 (45.8)	27 (50.9)	84 (45.2)	199 (45.4)	0.73
Shunt placement type					0.02
Endoscopic third ventriculostomy	104 (41.0)	4 (21.1)	28 (40.6)	72 (43.4)	
Ventriculoperitoneal	146 (57.5)	13 (68.4)	40 (58.0)	93 (56.0)	
Ventriculoatrial	2 (0.8)	1 (5.3)	0 (0.0)	1 (0.6)	
Other	2 (0.8)	1 (5.3)	1 (1.4)	0 (0.0)	
Location of fourth ventricle					0.04
Normal	286 (79.4)	15 (71.4)	69 (70.4)	202 (83.8)	0.01
Low	30 (8.3)	2 (9.5)	11 (11.2)	17 (7.1)	0.44
At foramen magnum	17 (4.7)	0 (0.0)	8 (8.2)	9 (3.7)	0.13
Below foramen magnum	27 (7.5)	4 (19.1)	10 (10.2)	13 (5.4)	0.04
Epidermoid cyst	31 (4.6)	0 (0.0)	7 (3.8)	24 (5.5)	0.16
Surgery for tethered cord	0 (0.0)	0 (0.0)	0 (0.0)	0 (0.0)	
Chiari decompression surgery	5 (0.7)	0 (0.0)	2 (1.1)	3 (0.7)	0.71
Shunt infection	9 (3.2)	0 (0.0)	2 (2.7)	7 (3.8)	0.61
Birthweight (g, median, IQR)	2500 (2050–2910)	1010 (830–1210)	2020 (1750–2220)	2770 (2480–3040)	<0.01
Dehiscence at fetal repair site					0.58
Intact	595 (93.3)	45 (95.7)	157 (91.8)	393 (93.6)	
Dehiscence	43 (6.7)	2 (4.3)	14 (8.2)	27 (6.4)	
Apnea	252 (37.2)	46 (86.8)	113 (60.8)	93 (21.2)	<0.01
Respiratory distress syndrome	181 (26.7)	41 (77.4)	76 (40.9)	64 (14.6)	<0.01
Pneumothorax	19 (2.8)	3 (5.7)	8 (4.3)	8 (1.8)	0.1
Patient ductus arteriosus	33 (4.9)	19 (35.9)	9 (4.8)	5 (1.1)	<0.01
Sepsis	36 (5.3)	15 (28.3)	10 (5.4)	11 (2.5)	<0.01
Necrotizing enterocolitis	2 (0.3)	1 (1.9)	0 (0.0)	1 (0.20	0.08
Intraventricular hemorrhage	12 (1.8)	5 (9.4)	3 (1.6)	4 (0.9)	<0.01
Periventricular leukomalacia	15 (2.2)	3 (5.7)	8 (4.3)	4 (0.9)	<0.01
Foot deformity	231 (34.1)	18 (34.0)	59 (31.7)	154 (25.2)	0.71

Abbreviation: GA, gestational age; IQR, interquartile range.

For patients with available CSF diversion data, the median GA at delivery in the <30‐week cohort was 27.4 weeks and the median latency from surgery to delivery was 3.3 weeks (Table 4). There was no statistical difference between the three cohorts for those receiving VP shunts, with rates of 50.9%, 45.2%, and 45.4%, respectively. Endoscopic third ventriculostomy was utilized more frequently in the later GA cohorts than in the <30‐week cohort. Normal location of the fourth ventricle was identified less frequently for the <30‐week cohort than the >34‐week cohort, and there was a higher rate of fourth ventricle location below the foramen magnum compared to later cohorts.

## 4 DISCUSSION

This study provides data on outcomes for very preterm birth following in utero spina bifida repair that can be used in the decision‐making process for the families who are considering in utero spina bifida repair and for the surgical team who must counsel potential in utero surgical candidates. The principal findings were that 9% of patients following in utero spina bifida repair deliver prior to 30 weeks of gestation, resulting in a 21% perinatal mortality rate. Perinatal mortality was associated with a short latency from surgery to delivery and an average GA at delivery of 25–26 weeks of gestation.

The findings from this study correspond with the MOMS trial and available post‐MOMS data, which found similar rates of delivery prior to 30 weeks and an overall mortality rate of 2.2% for the entire cohort [1, 2]. It is clear that delivery prior to 30 weeks of gestation is associated with significant neonatal morbidity [10, 11]. In addition, there are higher rates of maternal complications in the very preterm birth cohort following in utero spina bifida repair including chorioamnionitis, placental abruption, and need for maternal blood transfusion [12, 13]. Some complications occurred at higher rates in the later GA cohorts such as chorioamniotic membrane separation and a thin hysterotomy scar, which may suggest that these particular complications may take time to develop and do not immediately develop postoperatively. Future pregnancy considerations should also be included in initial counseling for women contemplating prenatal MMC repair. In a previous fMMC registry study, it was determined that the risk of uterine rupture or dehiscence in subsequent pregnancies after open fetal repair is significant, but similar to that reported for subsequent pregnancies after classical cesarean deliveries [6]. The data also demonstrate that CSF diversion rate reduction, specifically VP shunt rates, a major benefit of in utero spina bifida repair, appears preserved despite very early delivery. The VP shunt rate reduction remains consistent with the MOMS trial that demonstrated a 40% shunt rate in the prenatal surgery group and a 82% rate in the neonatal repair group. Further data accrual is necessary to determine the impact very preterm delivery has on the ability to achieve independent ambulation as well as on neurodevelopmental outcomes for these children, especially since premature birth can have significant impact on achieving developmental milestones.

Although there are many confounding variables, when comparing neonatal mortality and morbidity following in utero spina bifida repair to evaluable data from the Vermont Oxford Network, one of the largest neonatal outcome databases worldwide with extensive data on very low birth weight infants, the premature neonates in the fMMC cohort have comparable morbidity and mortality rates to neonates without MMC delivered at similar GAs. For example, for all neonates born between GAs of 25–27 weeks for delivery for the year 2020, ranges are ∼8%–20% for mortality, ∼10%–20% for sepsis, ∼8%–12% for intraventricular hemorrhage, and ∼5%–10% for necrotizing enterocolitis. Apnea and respiratory distress affect nearly all neonates born within this GA range [14]. These are similar to the rates reported in this cohort of neonates with spina bifida born prematurely after fetal surgery.

The preservation in the reduction of VP shunt in the <30 weeks’ delivery cohort may be explained by the early documentation of hindbrain herniation reversal identified during postoperative evaluation. Many centers perform fetal magnetic resonance imaging postoperatively as part of their postsurgical evaluation. Some perform this as early as 2 weeks after in utero spina bifida repair with high rates of documented hindbrain herniation reversal [15]. Delivery occurred on average 3.3 weeks after fetal surgery for those with VP shunt data in the <30‐week cohort, likely allowing enough time to establish the beneficial effects of prenatal surgery.

### 4.1 Clinical and research implications

The number of centers offering in utero spina bifida repair has expanded since the publication of the MOMS trial [16, 17], but the very preterm delivery rate and perinatal mortality rate has remained constant. Thus, despite expanding inclusion criteria and loosening exclusion criteria from the randomized clinical trial, there has not been an increase in these rates [18, 19, 20]. However, despite a decade passing since the MOMS trial was published, premature birth remains a significant complication of prenatal surgery. Further refinement in techniques such as a mini‐hysterotomy and the introduction of minimally invasive alternative approaches, including fetoscopic myelomeningocele closure have not demonstrated an improvement in these complication rates [21, 22, 23, 24]. For example, in a series of 300 cases of fetoscopic myelomeningocele repair, there was no difference in rates of delivery prior to 30 weeks for the fetoscopic group compared to the MOMS trial and post‐MOMS cohorts, despite a less invasive approach [25]. In fact, complications associated with preterm birth were higher in the fetoscopic group, including placental abruption, chorioamniotic membrane separation, premature rupture of membranes, and oligohydramnios. Although determining predictors of preterm birth was not a focus of this report, we evaluated a backward selected logistic model with maternal perioperative and postoperative variables with enough data in the registry for an analysis including surgical method (fetoscopic vs. open), nulliparity, prior uterine surgery, anterior placenta, lesion level, lesion type, maternal BMI, and GA at initial evaluation. Patients who underwent a fetoscopic procedure had 2.4 (95% CI, 1.2–4.6) increased odds of having a preterm delivery <30 weeks. Further data are required on CSF diversion rates following fetoscopic repair, as there were only 17 patients currently available in the registry. Regardless of surgical approach, families considering prenatal repair must be counseled on the rate of very preterm birth and contend with the potential for perinatal mortality and neonatal morbidity associated with this very real complication.

Dr. Michael Harrison, a pioneer in fetal surgery, stated that premature delivery is the Achilles heel of fetal surgery [26]. This remains true, and funding and research should be devoted to preventing preterm birth. In addition to attempts to limit complications by moving to fetoscopic approaches and improvement in instrumentation, there have been thus far unsuccessful attempts to reduce prematurity by attempts at sealing, plugging, and fixing amniotic membrane defects without clinical success. It has been suggested that future success may come from a better understanding of amniotic membrane biology and the use of tissue engineering leading to less invasive approaches to repair spina bifida thus reducing risks for premature delivery [27, 28, 29, 30]. There are limited data on performing fetal surgery at later gestational to avoid neonatal morbidity and mortality. In one study, fetoscopic repair was performed in 42 patients between 26 and 27 weeks of gestation with similar CSF diversion rates to those cases performed prior to 25 weeks of gestation [31].

### 4.2 Strengths and weaknesses

The use of the fMMC database for a retrospective analysis demonstrates notable strengths and weakness. The fMMC registry provides a large sample size, which increases statistical power and reliability of findings. The inclusion of multiple centers with differing practices from North America and Europe allows the capture of a broad representative patient population and enhances the generalizability of outcomes. The data represent actual clinical practice outside of randomized clinical trials and likely represents real‐world data.

There are also several weaknesses to the fMMC registry data. With many centers providing data, there can be significant variations in both data availability and entry, which can potentially impact quality and reliability. Accuracy of data entry is dependent on each specific center. The data cannot be delineated into center‐specific analyses and cannot consider variations in clinical practice among centers. The decision for VP shunt or endoscopic third ventriculostomy (ETV) was left largely to the provider and a standardized approach is lacking. As such, severe prematurity might have been seen as an increased surgical risk, possibly raising the bar to performing a VP shunt. There is a paucity of data regarding CSF diversion rates for very premature infants. In one study, it was determined that at least weight at the time of shunt placement does not change the rate of revision or outcome between infants weighing 2000–3000 g and low weight infants weighing less than 2000 g [32]. Long‐term follow‐up for these children remains challenging and problematic. For this study, there are limited data about rates of independent ambulation and neurodevelopmental outcomes, which is critical to better inform patients of the risks and benefits of prenatal surgery.

Finally, the data used for this project were collected retrospectively through EHRs. EHRs were designed for capturing health details relevant to billing, scheduling, and clinical record keeping, not research. Therefore, information important for research, like demographic variables and diagnostic codes, may be insufficiently or inaccurately captured in EHRs [33, 34, 35].

## 5 CONCLUSION

Very preterm birth following in utero spina bifida repair results in significant perinatal mortality and neonatal morbidity. Maternal morbidity is also significantly higher in this early delivery cohort. Despite very preterm birth, the reduction in VP shunt rates, a major benefit of in utero repair, appears to be preserved. Further data are required to analyze the impact on both independent ambulation and neurodevelopmental outcomes following prenatal myelomeningocele repair. Further research into reducing prematurity following fetal surgery continues to be of paramount importance.

## CONFLICT OF INTEREST STATEMENT

The authors declare no conflicts of interest.

## Data Availability

The data that support the findings of this study are available from fMMC registry. Restrictions apply to the availability of these data, which were used under license for this study. Data are available from the author(s) with the permission of fMMC registry.
